# Brain Activity Changes in Slow 5 and Slow 4 Frequencies in Patients With Optic Neuritis: A Resting State Functional MRI Study

**DOI:** 10.3389/fneur.2022.823919

**Published:** 2022-02-21

**Authors:** Kai Yan, Wen-Qing Shi, Ting Su, Xu-Lin Liao, Shi-Nan Wu, Qiu-Yu Li, Jing Yu, Hui-Ye Shu, Li-Juan Zhang, Yi-Cong Pan, Yi Shao

**Affiliations:** ^1^Department of Radiology, Hangzhou TCM Hospital Affiliated to Zhejiang Chinese Medical University, Hangzhou, China; ^2^Department of Ophthalmology, Jiangxi Centre of National Clinical Ophthalmology Institute, The First Affiliated Hospital of Nanchang University, Nanchang, China; ^3^Department of Ophthalmology, Massachusetts Eye and Ear, Harvard Medical School, Boston, MA, United States; ^4^Department of Ophthalmology and Visual Sciences, The Chinese University of Hong Kong, Hong Kong, Hong Kong SAR, China; ^5^Department of Acupuncture and Moxibustion, Hangzhou TCM Hospital Affiliated to Zhejiang Chinese Medical University, Hangzhou, China

**Keywords:** amplitude of low-frequency fluctuation, optic neuritis, resting state, functional magnetic resonance imaging, slow 5 and slow 4 frequencies

## Abstract

**Objective:**

We used the amplitude of low-frequency fluctuation (ALFF) method to investigate spontaneous brain activity in patients with optic neuritis (ON) in specific frequency bands.

**Data and Methods:**

A sample of 21 patients with ON (13 female and eight male) and 21 healthy controls (HCs) underwent functional magnetic resonance imaging (fMRI) scans in the resting state. We analyzed the ALFF values at different frequencies (slow-4 band: 0.027–0.073 Hz; slow-5 band: 0.01–0.027 Hz) in ON patients and HCs.

**Results:**

In the slow-4 frequency range, compared with HCs, ON patients had apparently lower ALFF in the insula and the whack precuneus. In the slow-5 frequency range, ON patients showed significantly increased ALFF in the left parietal inferior and the left postcentral.

**Conclusion:**

Our results suggest that ON may be involved in abnormal brain function and can provide a basis for clinical research.

## Introduction

Optic neuritis (ON) refers to all inflammatory lesions of the optic nerve, usually manifesting as acute or subacute vision loss with or without orbital pain, eye rotation pain, visual field defects or other clinical symptoms, and is the most common neurological disease leading to visual loss in young and middle-aged adults (1). The causes of optic neuritis are thought to include demyelinating diseases of the central nervous system, infectious diseases and immune diseases. A survey found that ON was the second most damaging disease among patients under 50 years of age after glaucomatous optic neuropathy (2). Worldwide, the annual incidence of unilateral optic neuritis is (0.94–2.18)/100,000, of which the annual incidence in the United Kingdom is 1/100,000 and that in Japan is 1.6/100,000 (3, 4). ON occurs predominantly in people aged 20–50, with an average age of onset of 36 years, and more than 70% of patients are female (5). Evidence suggests that there are racial and population differences in ON, which is more common among young, middle-aged, white females (6). Studies have shown that 80% of patients may have severe vision loss, and more than 60% of patients have monocular or binocular vision loss within 7.7 days after the first episode (7). The high incidence, young age of onset, high risk of blindness, and poor visual prognosis of ON have brought great physical and psychological burdens to patients and families, seriously affecting patients' quality of life. Its early diagnosis and treatment has become the focus of current research and the development of neuroimaging has provided a new method for the diagnosis of ON.

Magnetic resonance imaging (MRI) techniques have evolved rapidly to provide a non-invasive neuroimaging method that can assess functional and structural changes in the brain (8). Previous studies have demonstrated that synchronous brain activity is closely related to visual experience (9, 10). Resting state functional MRI (rsfMRI) was first proposed by Biswal (11) to assess consistent patterns of spontaneous fluctuation of blood oxygen level dependent (BOLD) signals during rest. These signals can be used to measure interhemispheric coordination (12).

The amplitude of low-frequency fluctuation is a widely used re-fMRI study method, which reflects the blood oxygen level-dependent (BOLD) signal of spontaneous neural activity in the low-frequency band (0.01–0.08 Hz) and can detect the intrinsic local activity of the brain. In recent years, domestic and international researchers have started to use sub-band ALFF analysis to study the correlation between neural activity and cognitive function in different neurological diseases by subdividing the frequency range of spontaneous brain activity, including slow-6 (0–0.01Hz), slow-5 (0.01–0.027Hz), slow-4 (0.027–0.073Hz), slow-3 (0.073–0.198Hz), slow-6 (0–0.01Hz), and slow-2 (0.198–0.250Hz). Among them, slow-2, 3 and 6 frequencies correspond to high frequency physiological noise interference, white matter signal and low frequency drift signal, respectively. And the study of slow-4 and slow-5 specific frequency bands can study the spatial distribution characteristics of brain regions with abnormal spontaneous functional activity in the brain in more depth, which in turn reduces the interference of noise in other frequency bands and increases the sensitivity of detecting abnormal activity in brain regions. RsfMRI and both functional and anatomic imaging have been applied to various ocular diseases (13–19).

The present study aims to determine whether spontaneous brain activity is related to the clinical characteristics of patients with ON. To detect the spontaneous brain activity of ON patients and healthy controls (HCs), we used the amplitude of low-frequency fluctuation (ALFF) technique to measure activity in different low frequency bands (slow 4 band: 0.027–0.073 Hz and slow 5 band: 0.01–0.027 Hz) and compared these between groups.

## Materials and Methods

### Participants

**Figure 1:**
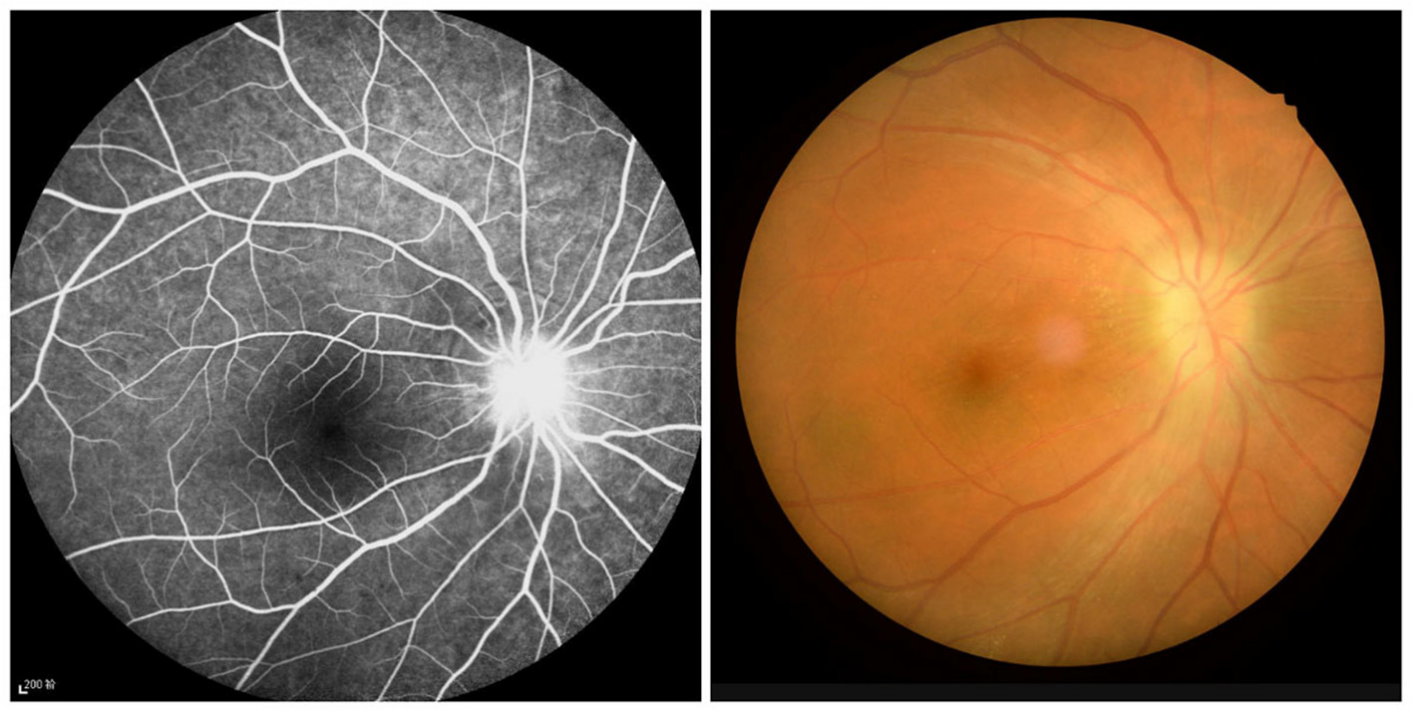
Example of optic neuritis observed using a FC and FFA. FC, fundus camera; FFA, fluorescence fundus angiography.

This study included 21 ON patients (13 females and eight males) admitted to the Ophthalmology Department of the First Affiliated Hospital of Nanchang University (Nanchang, China). The selection criteria for all ON patients were: (1) the presence of eye pain related to acute vision loss; (2) nerve fiber damage and abnormal vision (Figure 1); (3) pupil block or abnormal visual evoked potential; (4) no other apparent cause of acute vision loss; (5) no medication taken before the examination; (6) no drug, alcohol, or tobacco addictions; and (7) no history of organ transplantation.

In addition, 21 age-matched HCs (13 females and eight males) were recruited. The inclusion criteria for HCs were as follows: (1) no abnormalities in brain parenchyma; (2) unilateral bare eye vision≥1.0 (3) normal nervous system.

All participants are able to go through an MRI scan. We used logMAR (Logarithm of Mininal Angle Resolution) method to obtain the best-corrected visual acuity (BCVA) of both groups and mean hospital anxiety and depression scale (HADS) to get the association with ALFF values.

The study was approved by the ethics committee of the First Affiliated Hospital of Nanchang University, and all methods were applied in accordance with the Helsinki Declaration. The study design description was provided to the participants before they signed informed consent forms.

### MRI Parameters

All subjects underwent MRI scans in a 3-Tesla scanner (Trio, Siemens, Munich, Germany). They are constrained to the inspection area of the instrument and their heads are secured to prevent movement during the scanning process. During examination, the subjects remained awake, closed their eyes, and relaxed while avoiding focused thought. All scans were conducted by the same imaging physician, who observed the subjects until the process was completed successfully. Routine brain localization and T1 and T2 sequences were performed first. The specific parameters were: repeat time (TR) = 1,900 ms; echo time (TE) = 2.26 ms, thickness =1.0 mm, gap = 0.5 mm, acquisition matrix = 256 × 256, field of view (FOV) = 250 mm × 250 mm, and reversal angle = 90°. No substantial brain lesions were identified in the scanning process. RsfMRI data were acquired using a gradient-recalled echo sequence. The specific parameters were: TR = 2,000 ms, TE = 30 ms, thickness = 4.0 mm, gap = 1.2 mm, acquisition matrix = 64 × 64, flip angle = 90°, FOV = 220 mm × 220 mm. Data were collected continuously at 240 time points, and the scanning range included the whole brain.

### MRI Data Processing

Data were obtained from functional images using the MRIcro software package (www.MRIcro.com) after prefiltering. SPM8 (http://www.fil.ion.ucl.ac.uk/spm) and DPARSFA (http://rfmri.org/ DPARSF) software were used to preliminarily analyze data before conversion to the NIFTI format. During magnetization equilibration the first 10 time points were discarded. Subjects were excluded if they had 1.5 angular motion or more than 1.5 mm maximum shift in the x, y, or z direction during the fMRI examination. After the head motion correction, the fMRI images were spatially normalized to the Montreal Neurological Institute space criteria using the standard echo-planar imaging template and resampling the images at a resolution of 3 mm × 3 mm × 3 mm. On the basis of the above, the signals of slow-4 and slow-5 were extracted separately using band-pass filtering oscilloscope, and the ALFF values were calculated and analyzed by REST software (http://sourceforge.net/projects/testing-fmri).

### Statistical Analysis

Student's *t*-test was used to compare ALFF data between groups (20). A two-way analysis of variance and *post-hoc* tests were used to compare interaction s between groups and frequency bands. Based on Gaussian random field theory [z > 2.3, crowd >40 voxels, *P* < 0.01, false detection rate (FDR) corrected], we regulate the voxel offset (*P* < 0.05) as the statistical premise for comparisons.

### Brain-Behavior Correlation Analysis

Pearson correlation analysis was used to look for associations between ALFF and clinical level using a threshold of *P* < 0.05 for statistical significance.

## Results

### Clinical Characteristics of the Sample

No significant differences were found in age (*P* = 0.821), weight (*P* = 0.652), height (*P* = 0.634), or BMI (*P* = 0.963) between the two groups. Significant differences in best-corrected VA-right (*P* < 0.001) and best-corrected VA-left (*P* < 0.001) between the ON and HC groups. Details are shown in Table 1.

**Table 1:** Clinical characteristics of patients between ON and HC groups.

**Characteristics**	**ON**	**HCs**	***t*-value**	***p*-values**
Male/female	813	813	NA	NA
Age (years)	44.83 ± 10.71	45.83 ± 11.38	−0.222	0.821
Weight (kg)	57.08 ± 7.30	58.85 ± 5.85	−0.463	0.652
Height (cm)	160.81 ± 9.31	161.38 ± 6.28	−0.485	0.634
BMI (kg/m^2^)	21.13 ± 1.62	21.17 ± 1.27	−0.056	0.963
Duration of ON (days)	4.67 ± 3.26	NA	NA	NA
Duration from onset of ON to rs-fMRI scan (days)	5.42 ± 2.94	NA	NA	NA
Best-corrected VA, right	0.25 ± 0.32^*^	1.30 ± 0.31	−8.138	<0.001
Best-corrected VA, left	0.85 ± 0.52^*^	1.28 ± 0.32	−2.481	<0.001

*Independent t-tests comparing the two groups (*

* *P <0.05) represented statistically significant differences). ON, optic neuritis; HCs, healthy controls; NA, not applicable; BMI, body mass index; rs-fMRI, resting-state functional magnetic resonance; VA, visual acuity*.

### Changes in ALFF in Different Frequency Bands

**Figure 2:**
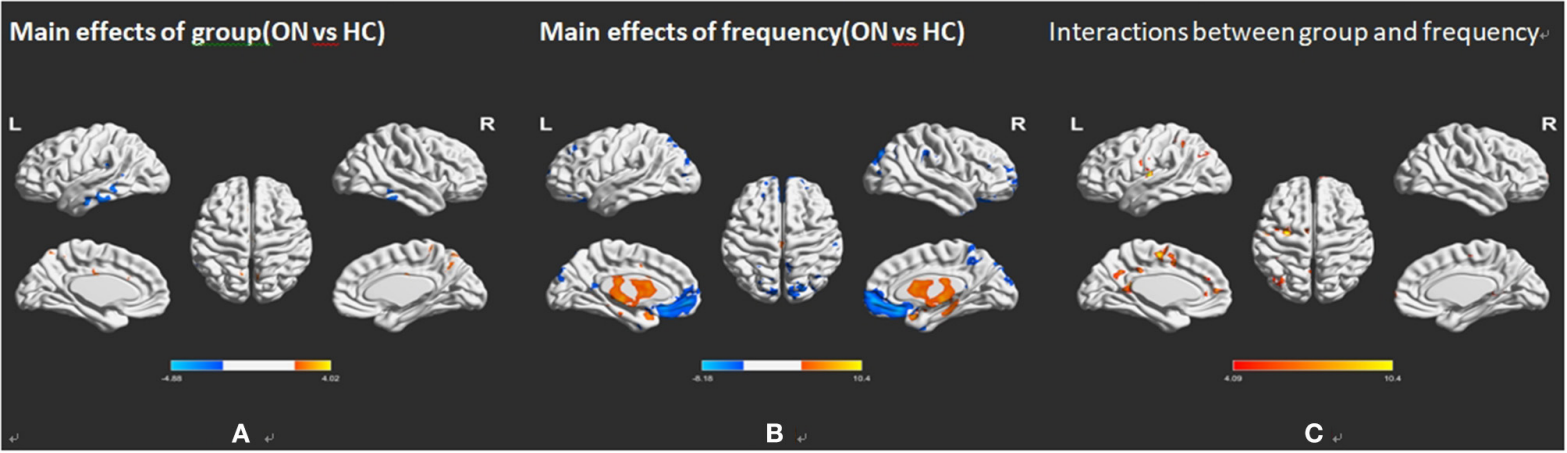
Frequency-associated ALFF analyses between the ON and HC groups. **(A)** Main effects of group, **(B)** main effects of frequency band, and **(C)** their interactions, with Gaussian random field theory correction, voxel-level *P* < 0.01 and cluster-level *P* < 0.05. Compared with HC, red represents the brain areas with increased AlFF, and blue represents the brain areas with decreased AlFF in patients with ON. ALFF, amplitude of low-frequency fluctuation; ON, optic neuritis; L, left; HC, healthy control; R, right.

As shown in Figure 2A and Table 2, he two-way repeated-measures ANOVA was applied to explore main effects between groups. There were apparently increased ALFF (ON > HCs) in the right cerebellum, the right temporal inferior (temporal inf), the left temporal inf, and the left parietal inferior (parietal inf) and remarkably decreased ALFF (ON < HCs) in the left cingulum anterior (cingulum ant) and the right cingulum ant in brain regions with a main effect of group.

**Table 2:** Frequency-associated ALFF differences, with Gaussian random field theory correction, voxel-level *P* < 0.01 and cluster-level *P* < 0.05.

**Brain area**	**BA**	**Peak (MNI)**			**Peak voxels**	***T*-value**
		**X**	**Y**	**Z**		
Main effects of group						
Left cingulum ant	24	−6	10	20	89	13.11
Right cingulum ant	24	0	−12	30	83	23.11
Right cerebellum	/	18	−87	−45	800	13.67
Right temporal inf	20	45	−30	−21	117	10.02
Left temporal inf	21	−57	−12	−30	331	18.68
Left parietal inf	40	−54	−36	42	82	18.31
Main effects of frequency band						
Right rectus	11	9	27	−18	930	−1.88
Right occipital sup	19	24	−84	39	106	−6.2
Left caudate	24	−21	−36	12	3,044	10.4
Left temporal pole sup	38	−3	12	−24	145	7.08

*ALFF, amplitude of low-frequency fluctuation; BA, Brodmann area; MNI, Montreal neurological institute*.

In several brain regions (Figure 2B; Table 2), the ALFF differed significantly between frequency bands, including increased ALFF of the right rectus gyri (slow-5 > slow-4), right supraoccipital segment (right supraoccipital) and decreased ALFF (Figure 2B; Table 2). Slow-5 < slow-4 in left caudate and left temporal pole sup. No obvious interaction was found between groups and frequency bands (Figure 2C).

**Figure 3:**
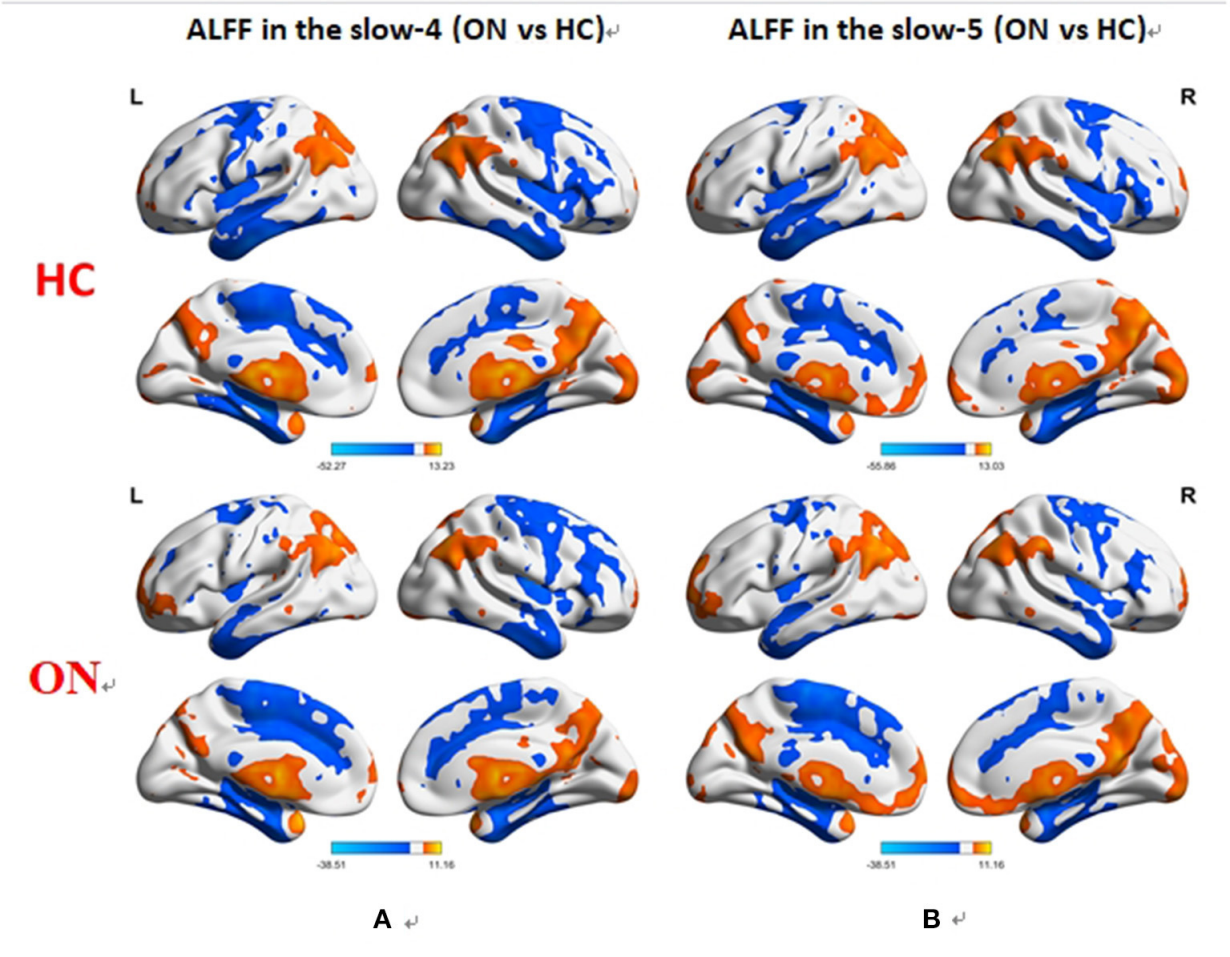
Difference in the ALFF values in the brain between the ON and HC groups across slow-4 **(A)** and slow-5 **(B)** frequencies, with Gaussian random field theory correction, voxel-level *P* < 0.01 and cluster-level *P* < 0.05. Compared with HC, red represents the brain areas with increased AlFF, and blue represents the brain areas with decreased AlFF in patients with ON. ALFF, amplitude of low-frequency fluctuation; ON, optic neuritis; L, left; HC, healthy control; R, right.

**Figure 4:**
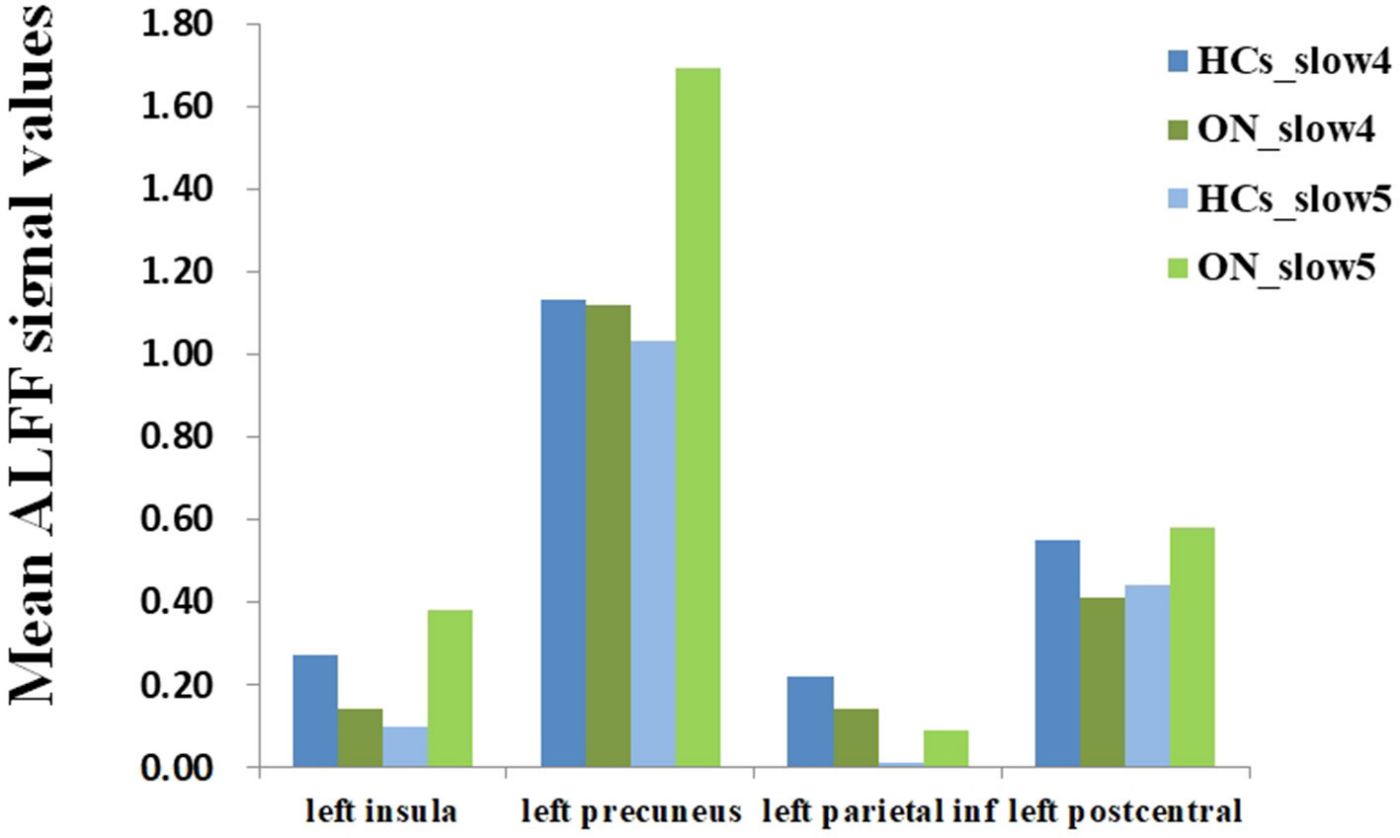
The mean ALFF values of altered brain regions between the ON and HCs. ALFF, amplitude of low-frequency fluctuation; ON, optic neuritis; HCs, healthy controls.

No bidirectional changes were detected by *post hoc t*-tests in ALFF across slow-4 and slow-5 frequencies. Significantly lower ALFF values were found in the left insula and the left precuneus in the slow-4 band, but significantly higher ALFF values in the left parietal inf and the left postcentral in the slow-5 band (Figure 3; Tables 3, 4). The mean ALFF values between the ON patients and HCs are represented in Figure 4.

**Table 3:** In the slow-4 band (0.027–0.073 Hz), ALFF differences between the PHN and HC groups with Gaussian random field theory correction, voxel-level *P* < 0.01 and cluster-level *P* < 0.05.

**Brain area**	**BA**	**Peak (MNI)**			**Peak voxels**	***T*-value**
		**X**	**Y**	**Z**		
ON < HCs						
Left insula	13	−39	−12	12	173	23.01
Left precuneus	31	−3	−54	33	111	12.67

*ALFF, amplitude of low-frequency fluctuation; ON, optic neuritis; HCs, healthy controls; BA, Brodmann area; MNI, Montreal neurological institute*.

**Table 4:** In the slow-5 band (0.001–0.027 Hz), ALFF differences between the PHN and HC groups with Gaussian random field theory correction, voxel-level *P* < 0.01 and cluster-level *P* < 0.05.

**Brain area**	**BA**	**Peak (MNI)**			**Peak voxels**	***T*-value**
		**X**	**Y**	**Z**		
ON>HCs						
Left parietal inf	40	−30	−48	54	109	18.68
Left postcentral	6	−33	−21	45	417	18.31

*ALFF, amplitude of low-frequency fluctuation; ON, optic neuritis; HCs, healthy controls; BA, Brodmann area; MNI, Montreal neurological institute*.

### Correlation Analysis

**Figure 5:**
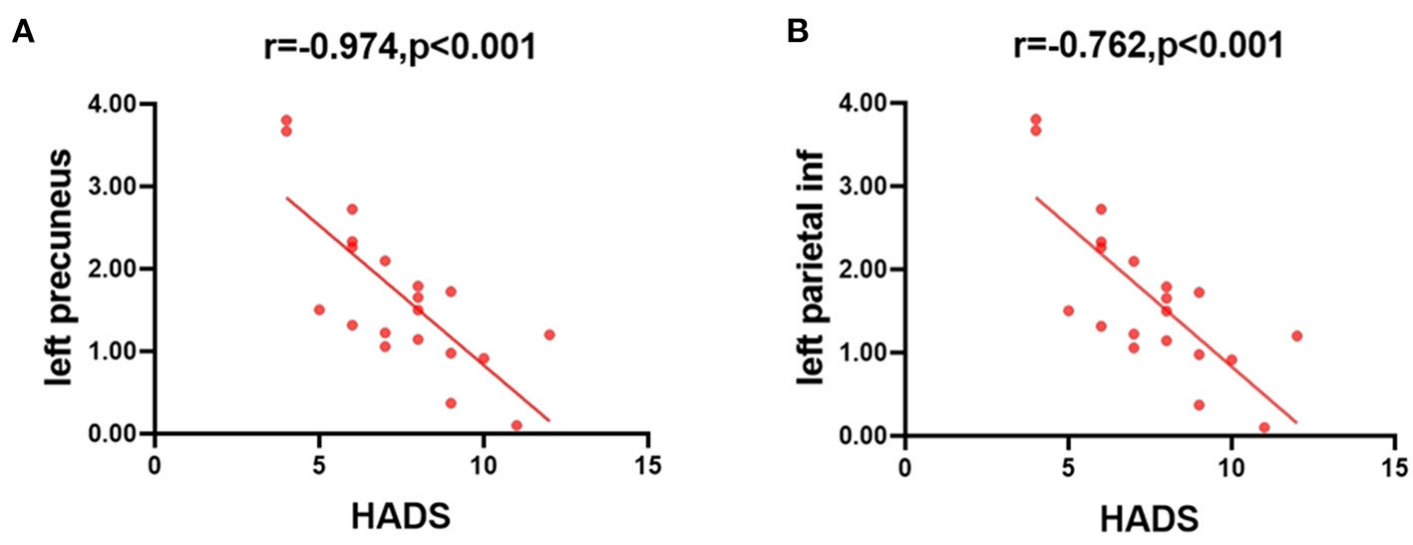
Correlations between the mean ALFF values and the clinical behaviors. **(A)** The HADS scores showed a negative correlation with the ALFF value of the slow-4 band in the left precuneus (*r* = −0.974, *P* < 0.001), and **(B)** the HADS scores showed a negative correlation with the ALFF value of the slow-4 band in the left parietal inf (*r* = −0.762, *P* < 0.001). ALFF, amplitude of low-frequency fluctuation; HADS, hospital anxiety and depression scale.

In the patients with ON, the mean HADS scores negatively correlated with the ALFF values of the slow-4 band in the left precuneus (*r* = −0.974, *P* < 0.001), and in the left parietal inf (*r* = −0.762, *P* < 0.001) (Figure 5).

## Discussion

ALFF is a common clinical method reflecting the relationship between various clinical diseases and corresponding brain regions, as shown by many previous studies (Table 5).

**Table 5:** Amplitude of low-frequency fluctuation method applied in ophthalmological diseases.

**Reference**	**Disease**
Huang et al. (13)	Primary angle-closure glaucoma
Tan et al. (14)	Congenital comitant strabismus
Li et al. (15)	Monocular blindness
Pan et al. (18)	Acute eye pain
Shi et al. (16)	Corneal ulcer
Kang et al. (17)	Retinal detachment
Wu et al. (19)	Retinal vein occlusion

### Main Effect of the Groups

ALFF values were significantly higher in ON patients than HCs in the right cerebellum, the right temporal inf, the left temporal inf, and the left parietal inf, and lower than HCs in the left cingulum ant and the right cingulum ant.

The cerebellum is responsible for balance, cognitive tasks and motor control. We have shown using fMRI that the cerebellum is involved in cognition and memory (21). Previous studies have linked cerebellar dysfunction to schizophrenia (22), bipolar disorder (23), and depression (24) and demonstrated higher cerebellar activation in patients with primary progressive multiple sclerosis (MS) than in healthy controls (25–27). Similarly, we found that the ALFF value of the right cerebellum is higher in ON patients than in controls, which may reflect functional compensation for neural damage. In the resting state, the default model network (DMN) is continuously activated in the brain and is associated with many conscious activities (28–32). In about 20% of MS patients, ON is the most important clinical feature (33). Previous research has shown (34) that DMN connectivity of the anterior cingulate cortex of MS is weaker than in controls and that the converse is true in the posterior cingulate cortex, with relatively strong connectivity in MS. In line with these findings, the present study found decreased ALFF in patients with ON in the bilateral cingulum ant and left precuneus, and increased ALFF in the bilateral temporal inf and left parietal inf. ALFF may play vital roles in understanding functional reorganization in ON patients. Therefore, changes in the ALFF values of the bilateral cingulate gyrus and the left anterior gyrus may be explained by the DMN damage caused by ON, and the abnormalities of the bilateral temporal lobe and left parietal lobe inf may be related to the stability of the network.

### Main Effect of Frequency Band

Higher ALFF was found at slow-5 than at slow-4 frequency range in the right rectus, and right occipital sup and the converse, with lower ALFF at slow-5 than at slow-4, in the left caudate and left temporal pole sup. Various neurophysiological mechanisms give rise to a range of oscillatory bands with a correspondingly wide range of physiological functions (35). Consistent with previous studies, we found abnormal amplitudes in the frontal, occipital and parietal cortex in ON (35), with the slow 4 range dominating the ventral prefrontal cortex (36).

### Frequency-Dependent Alterations in ALFF in On Patients

We found bidirectional changes of ALFF both in the ON and HC groups across slow-4 and slow-5 frequency bands, providing new insight into frequency-dependent alterations of ON patients. Excessively secondary to ALFF decreased in insula and the punch precuneus gyrus in the slow-4 band, but ALFF values were seriously upper in the awaken parietal inf and the talk area over postcentral gyrus in the slow-5 band.

The insula is located in the depths of the lateral sulcus, (37) is anatomically related to the frontal, parietal, and temporal lobes (38) and plays vital roles in consciousness, bodily impulses, and in controlling and suppressing natural impulses (39, 40). Insular dysfunction has also been linked to diseases including Alzheimer's disease (41) and epilepsy (42). Previous studies have demonstrated abnormal activation of the insula in ON patients (43, 44). Based on these findings, we infer that ON may be involved in insula dysfunction.

**Figure 6:**
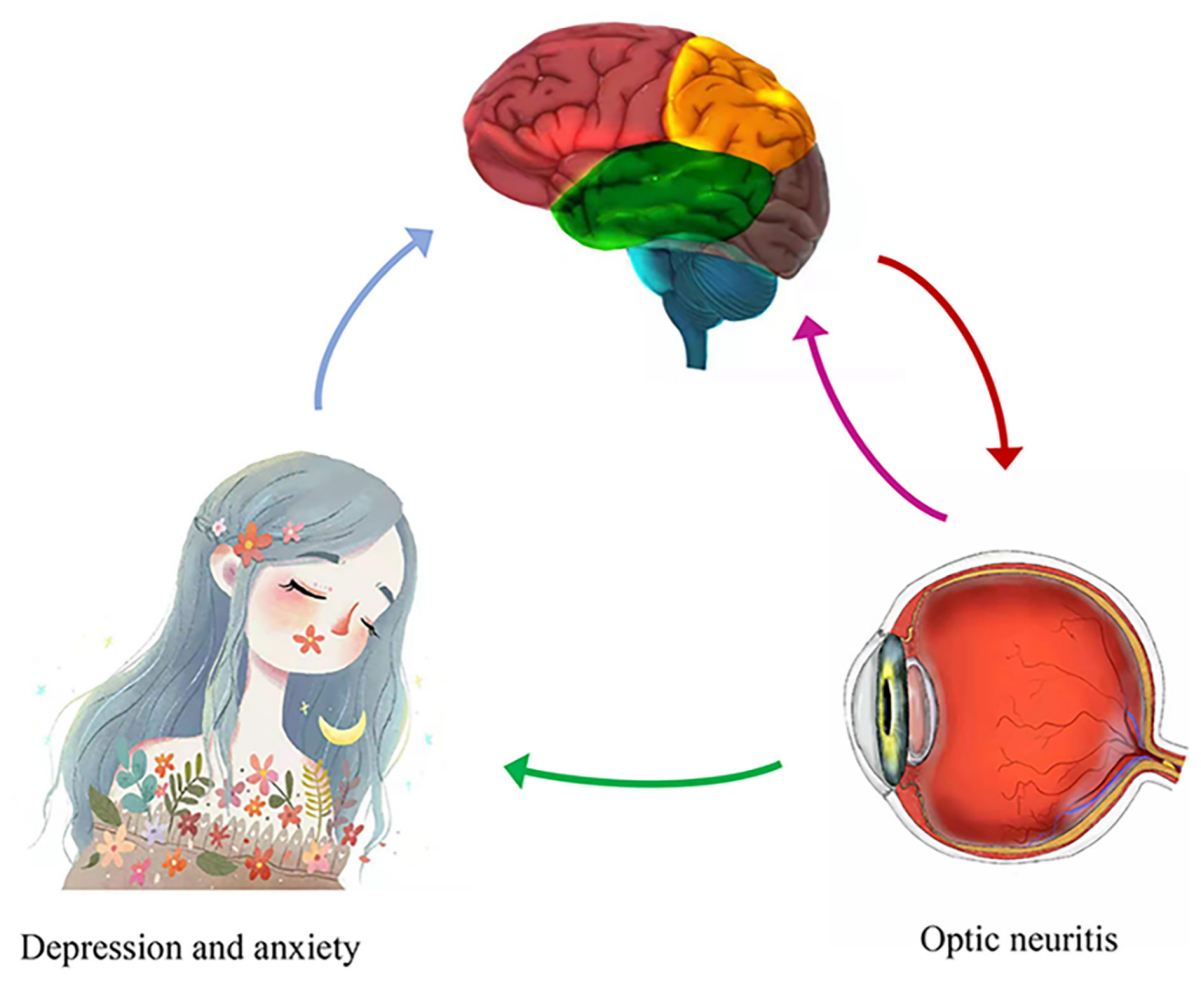
Correlations between mean ALFF values and behavioral performance. Compared with the HCs, the ALFF value of the left precuneus was decreased in ON. And the patients with ON exhibited with more depression and anxiety. ALFF, amplitude of low-frequency fluctuation; HCs, healthy controls; ON, optic neuritis.

Brain regions are associated with the DMN (45). It is increasingly evident that DMN vulnerability plays an important part in depression and anxiety (46). The present study found lower ALFF in the left precuneus in patients with ON which indicates that ON might lead to DMN damage (Figure 6).

The subparietal lobule participates in visual recognition (47). It is also related to diseases such as schizophrenia (48) and Alzheimer's disease (49). In our study, abnormality of left parietal atrial fibrillation in patients with ON may reflect compensation for abnormal visual function.

Located in the primary somatosensory cortex, the postcentral gyrus is associated with information processing triggered by tactile stimulation (50). The primary somatosensory cortex is involved in the perception of pain (51). Previous research has demonstrated reduced white matter in the postcentral gyrus in patients with neuromyelitis optical (52). In addition, the sensorimotor system of patients with optic neuromyelitis is impaired (53). In the present study, we demonstrated increased ALFF values in the left postcentral gyrus in patients with ON, which may reflect compensation for sensorimotor dysfunction (Table 6).

**Table 6:** Brain regions alteration and its potential impact.

**Brain regions**	**Experimental result**	**Brain function**	**Anticipated results**
Insula	ON < HCs	Involved in consciousness, bodily impulses, and controlling and suppressing natural impulses	Abnormal activation of areas in the insula, diseases such as Alzheimer's disease and epilepsy
Precuneus	ON < HCs	Part of the default model network	Depression and anxiety
Parietal inf	ON>HCs	Part of the default model network, correlated with visual word recognition	Depression and anxiety, reflect compensation of the visual function
Postcentral	ON>HCs	Associated with information processing of tactile stimuli	Compensate the impairment of the sensorimotor dysfunction

*ON, optic neuritis; HCs, healthy controls*.

## Limitations

Our research has limitations. First, the sample size is relatively small. In addition, the correlation between clinical characteristics of ON and ALFF values require further investigation. Thus, we are looking forward to designing more experiments to further elucidate the underlying molecular mechanisms.

## Conclusions

In summary, our study reveals abnormal spontaneous activities in regional brain areas in ON, and may provide insight into the underlying pathogenetic mechanism.

## Data Availability Statement

The raw data supporting the conclusions of this article will be made available by the authors, without undue reservation.

## Ethics Statement

The study was approved by the Ethics Committee of the First Affiliated Hospital of Nanchang University, and all methods were applied in accordance with the Helsinki Declaration. The patients/participants provided their written informed consent to participate in this study. Written informed consent was obtained from the individual(s) for the publication of any potentially identifiable images or data included in this article.

## Author Contributions

KY, Y-CP, and W-QS performed the experiments and collected the data. TS, X-LL, and L-JZ designed the current study. S-NW, Q-YL, H-YS, JY, and YS given final approval of the version to be published. KY wrote the manuscript. All authors made substantial contributions to this research, read, and approved the final manuscript.

## Funding

This research was supported by the National Natural Science Foundation of China (Nos. 81660158, 81160118, 81400372, 81460092, and 81500742), Natural Science research Foundation of Guangdong Province (Nos. 2017A030313614, 2017A020215187, and 2018A030313117), and Medical Science Foundation of Guangdong Province (No. A2016184).

## Conflict of Interest

The authors declare that the research was conducted in the absence of any commercial or financial relationships that could be construed as a potential conflict of interest. The reviewer HL declared a shared parent affiliation with several of the authors, W-QS, S-NW, Q-YL, H-YS, L-JZ, Y-CP and YS at the time of review.

## Publisher's Note

All claims expressed in this article are solely those of the authors and do not necessarily represent those of their affiliated organizations, or those of the publisher, the editors and the reviewers. Any product that may be evaluated in this article, or claim that may be made by its manufacturer, is not guaranteed or endorsed by the publisher.
